# Genetic risk of chronic pain conditions associated with risk of suicide death through an integrative analysis of EHR and genomics data

**DOI:** 10.1038/s41398-026-03861-6

**Published:** 2026-02-16

**Authors:** Seonggyun Han, Emily DiBlasi, Eric T. Monson, Andrey A. Shabalin, Lisa Baird, Danli Chen, Dirga Lamichhane, Doug Tharp, Elliott Ferris, Zhe Yu, W. Brandon Callor, Michael J. Staley, Qingqin S. Li, Virginia Willour, David K. Crockett, Karen Eilbeck, Amanda V. Bakian, Brooks R. Keeshin, Akiko Okifuji, Hilary Coon, Anna R. Docherty

**Affiliations:** 1 Department of Psychiatry & Huntsman Mental Health Institute, University of Utah School of Medicine, Salt Lake City, UT USA; 2 Department of Geography, University of Utah, Salt Lake City, UT USA; 3 Department of Neurobiology, University of Utah School of Medicine, Salt Lake City, UT USA; 4 Pedigree & Population Resource, Huntsman Cancer Institute, University of Utah, Salt Lake City, UT USA; 5 Office of the Medical Examiner, Utah Department of Health and Human Services, Salt Lake City, UT USA; 6 Neuroscience Therapeutic Area, Janssen Research & Development LLC, Titusville, NJ USA; 7 Department of Psychiatry, University of Iowa, Iowa City, IA USA; 8 Department of Veterans Affairs, Iowa City Health Care System, Iowa City, IA USA; 9 Clinical Analytics, Intermountain Health, Salt Lake City, UT USA; 10 Department of Biomedical Informatics, University of Utah School of Medicine, Salt Lake City, UT USA; 11 Department of Pediatrics, University of Utah, Salt Lake City, UT USA; 12 Department of Public Health and Caring Science, Child Health and Parenting (CHAP), Uppsala University, Uppsala, Sweden; 13 Departments of Anesthesiology and Psychology, University of Utah School of Medicine, Salt Lake City, UT USA

**Keywords:** Genomics, Psychiatric disorders, Comparative genomics

## Abstract

Chronic pain represents heritable conditions linked to suicide death. It has been suggested that a shared genetic predisposition may contribute to this relationship, but there has not yet been a comprehensive assessment of genetic and clinical overlaps of different types of chronic pain with suicide death. Here, we integrated whole-genome sequencing and electronic health records from 986 unrelated individuals of European ancestry who died by suicide in the Utah Suicide Mortality Research Study and 415 ancestrally-matched population controls selected for absence of disease. Polygenic scores (PGSs) for seven distinct types of chronic pain were calculated and tested in the suicide cohort. We observed significant positive associations of PGSs for multisite chronic pain (PGS_MCP_) and chronic widespread pain (PGS_CWP_) with suicide mortality. Sex-stratified analyses showed elevations in both males and females. Pain diagnosis-stratified analyses revealed associations with suicide death regardless of chronic pain diagnoses. Follow-up tests of PGSs for more specific pain conditions showed additional associations with suicide death for: 1) monoarticular arthritis, 2) back pain, and 3) chronic inflammatory demyelinating polyneuropathy across all suicide death individuals, and 4) irritable bowel syndrome within males only. In a multiple logistic regression test of all chronic pain PGSs associating suicide death status, four types of pain remained uniquely associated with suicide death, highlighting distinct subgroups within suicide death: some attributed to MCP and CWP, and others associated with monoarticular arthritis or chronic inflammatory demyelinating polyneuropathy. This cohort study reports associations between suicide death and PGSs from various pain conditions, regardless of sex or chronic pain diagnosis, suggesting that combining genetic and clinical risk factors may better identify genetic overlap, causal directions, and/or specific gene pathways.

## Introduction

Suicide is a significant global public health concern [1]. Suicide death is significantly heritable, genetically complex, and phenotypically heterogeneous. It is influenced by multifactorial risks, including physical and mental health conditions. Psychiatric conditions have been extensively studied in relation to suicide death, yet much less is known about the interacting mechanisms of how many other debilitating clinical conditions contribute to suicide death [2]. Importantly, many individuals who die by suicide have no documented psychiatric conditions, with recent findings indicating that pre-morbid physical health problems are also prevalent in suicide death [3–5]. This has underscored the importance of a comprehensive understanding of other clinical comorbidities of suicide death for developing effective prevention strategies.

Chronic pain, often considered within the spectrum of neurological disorders, is a major risk factor for suicide death even after accounting for co-morbid psychiatric conditions [6], with suicide risk increasing in individuals with chronic pain [7]. Previous epidemiological studies have supported that risk of suicide death is elevated in individuals with chronic pain compared to the general population [8–13]. For example, a recent study revealed that individuals with chronic pain have a higher prevalence of suicide, as shown through a phenome-wide comparison study between suicide deaths and living individuals with chronic pain diagnoses [14]. In addition, prior research has observed sex differences in both chronic pain and suicide [5, 15–17]. For example, a recent study found that pain site and extent associated with suicide risk vary significantly by sex, underscoring the importance of sex-stratified analyses to capture sex-specific heterogeneity in the pain-suicide relationship [18]. However, at present, few studies have examined genetic relationships of suicide with chronic pain, limiting our understanding of suicide risk within the context of chronic pain conditions [7]. Chronic pain and suicide death are complex, polygenic phenotypes with substantial individual-level differences. Each is significantly heritable [19, 20]. Underlying biological mechanisms, such as genetic liability, could be shared between suicide death and chronic pain [21]. For example, genetic risk for chronic pain is associated with several molecular pathways, including immune, neural, and dopaminergic systems, that are also significantly linked to suicide risk [22–24]. Notably, a recent population-based twin study suggested that the association of suicide with chronic pain may be driven by an underlying shared genetic etiology rather than a secondary phenotypic effect of chronic pain conditions on suicidality [21]. Therefore, shared genetic predispositions between chronic pain and suicide death may contribute substantially to their clinical comorbidities.

Polygenic scores (PGS) can be useful for classifying individuals by genetic risk and for calculating common variant genetic correlations among health-related phenotypes [25–29]. Since both chronic pain and suicide death are substantially polygenic, PGS analysis can be used to evaluate the overlapping genetic risk of complex chronic pain types with suicide death [20, 30–33]. Here, we comprehensively evaluated whether polygenic scores for several pain conditions are associated with suicide death case-control status with or without the presence of a clinical diagnosis of a pain condition. Analyses integrated genomic data and electronic health records (EHR) of a large cohort of individuals who died by suicide, and who additionally have a significant extended family risk of suicide mortality [34].

## Methods

**Fig. 1:**
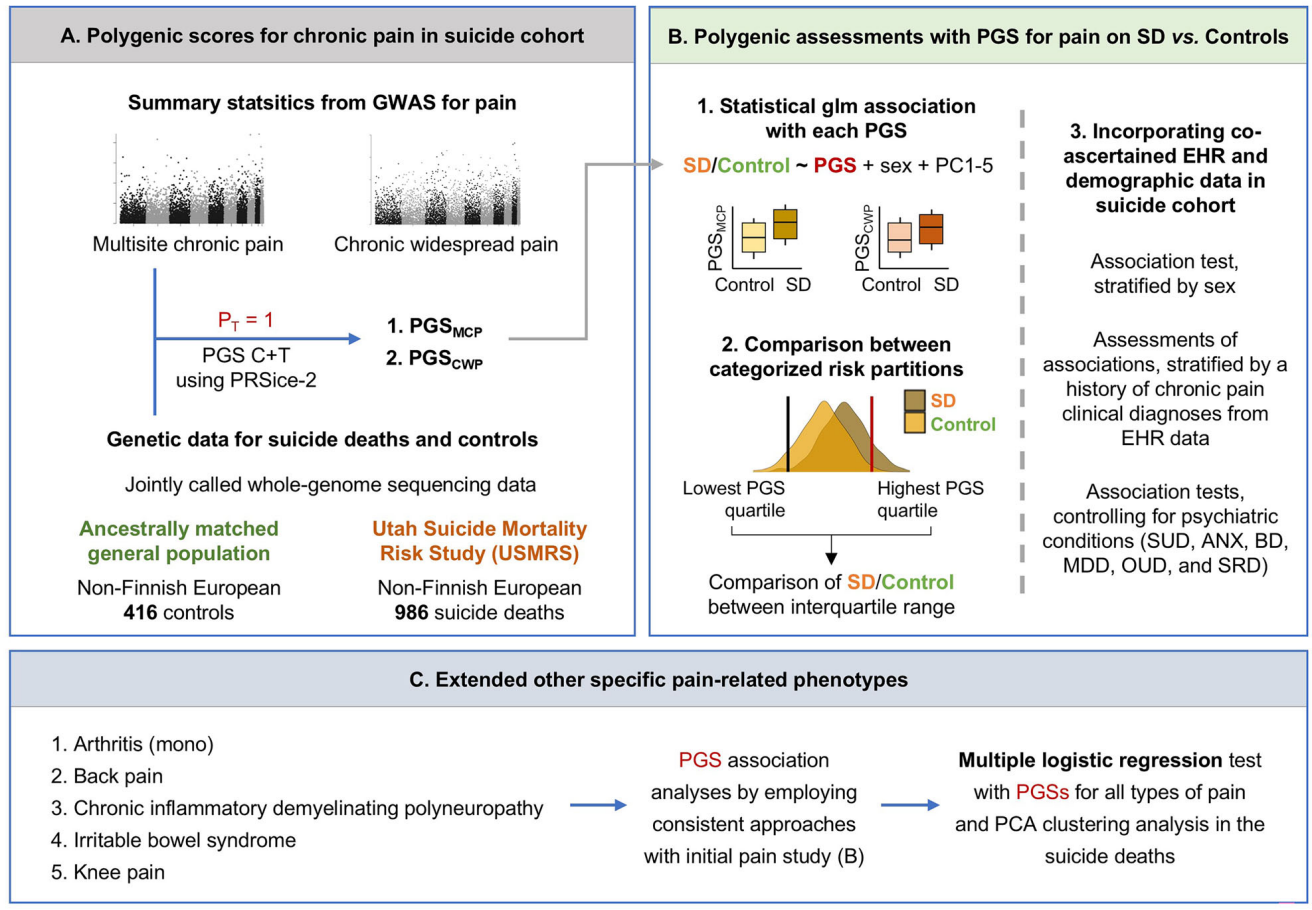
Overall research design of the PGS association analysis. **A** Summary statistics obtained from large-scale GWAS initially for two types of pain (e.g., multisite chronic pain and chronic widespread pain) were investigated to determine PGSs in our suicide cohort (n = 416 and 986 for controls and suicide death (SD), respectively. PRSice-2 was implemented for generating PGSs with threshold P = 1.0 incorporating all genomic information. **B** Generalized logistic regression was run to determine significance of associations of PGS with SD for each type of pain, adjusted for sex and ancestry principal components (PCs; PC1-PC5). In addition, comparisons of prevalent (SD) within the interquartile range were conducted: lowest quartile of PGS (individuals in first quartile) *vs*. intermediate quartile of PGS (those in second and third quartiles) and lowest quartile *vs*. highest quartile (those in fourth quartile). Furthermore, co-ascertained EHR and demographic data were incorporated. **C** Extended analyses of five additional pain-related phenotypes, monoarticular arthritis, back pain, chronic inflammatory demyelinating polyneuropathy, irritable bowel syndrome, and knee pain, were subjected to further analysis in alignment with the initial two pain PGS association study. This approach aimed to elucidate genetic relationships between a broader spectrum of pain conditions and SD risk. A multivariate association test employing PGSs for all pain types was performed to identify independent pain phenotypes associated with SD risk. SUD: substance use disorders; ANX: anxiety; BD: bipolar disorder; MDD: major depressive disorder; OUD: opioid use disorders; SRD: sleep related disorders.

An overview of the research design is depicted in Fig. 1.

### Utah suicide death cohort phenotypic electronic health records

The Utah Suicide Mortality Research Study (USMRS) has built a comprehensive population-based biobank of postmortem suicide deaths, encompassing DNA samples from over 8500 individuals, which are linked to extensive EHR data. The suicide samples are population-ascertained and established through a longstanding collaboration with the centralized Utah Office of the Medical Examiner (OME) in the State of Utah. DNA materials have been extracted from whole blood using state-of-the-art methods (https://ctsi.utah.edu/cores-and-services/ctrc/dna-extraction-facility). In the USMRS, the OME securely transfers suicide death data to the Utah Population Database (UPDB, https://uofuhealth.utah.edu/huntsman/utah-population-database), a comprehensive state-wide repository containing demographic and two decades of health records of over 12 million individuals. Previous work has extensively described data collection and the secure transfer of de-identified data in the USMRS to the research team [35].

### Identification of suicide deaths with clinical chronic pain

Approximately 85% of USMRS suicide deaths have EHR diagnostic data, specifically International Classification of Diseases (ICD) diagnostic codes (ICD-9; https://www.cdc.gov/nchs/icd/icd9.htm, ICD-10; https://www.cdc.gov/nchs/icd/icd10cm.htm). EHR diagnostic data includes all inpatient encounters, emergency department, and ambulatory care encounters state-wide, as well as outpatient encounters from the two largest clinical data providers in the state, University of Utah Healthcare and Intermountain Health. Medical diagnostic codes were aggregated via phecodes. Phecodes represent one validated way to define phenotypes using EHR data and reflect lifetime diagnoses of medical conditions [36]. ICD 9 Phecode Map 1.2 was used to aggregate ICD 9 codes and ICD 10 CM Phecode Map 1.2 beta was used to aggregate ICD 10 codes [37]. The presence of at least one ICD diagnostic code in the “Chronic pain” phecode (338.2; https://phewascatalog.org/), was used to define individuals with chronic pain diagnoses (Supplementary Table S1).

### Whole-genome sequencing data and electronic health records in the Utah Suicide Mortality Research Study

The genomic data include a whole-genome sequencing dataset of 986 European ancestry suicide deaths with electronic health data selected for extended family risk of suicide, and 415 (203 males and 212 females) ancestry-matched, population controls selected for absence of disease, as described in a previous study [35]. The whole-genome sequencing data on suicide deaths was generated using Illumina next-generation sequencing technology, ensuring an average read depth of at least 20x. Suicide death samples were biologically unrelated with >90% non-Finnish ancestry. Controls included 332 individuals from the 1000 Genomes Project cohort [38], 61 individuals from a Utah healthy longevity study [39], and 22 individuals from the multigenerational Centre d’Etude du polymorphisme humain [40]. All controls were confirmed to be unrelated and of European ancestral admixtures with pairwise identity-by-descent analysis and genetic ancestry principal component analysis. Genomic data from suicide and control samples were jointly called. More detailed descriptions of the processing steps, including quality control, mapping, variant calling, ancestry estimation, and control data, have been previously documented [35].

### Calculation of PGS for general chronic pain types in suicide deaths

We first calculated PGS in whole-genome sequencing samples focusing on two general types of chronic pain: multisite chronic pain (MCP) [41]; PGS_MCP_ and chronic widespread pain (CWP) [42]; PGS_CWP_. This estimation was based on publicly-available summary statistics obtained from multiple large-scale published genome-wide association studies (GWASs). The prior MCP study included ~380,000 UK Biobank European participants, assessing the sum of body sites with chronic pain lasting at least three months, and performed a GWAS treating this sum as a linear quantitative trait. Participants reporting pain “all over the body” were excluded to avoid including individuals with CWP [41]. The previous CWP GWAS included 249,843 participants of European ancestry from UK Biobank, comprising cases with pain all over the body lasting for at least three months [42]. The whole-genome sequencing data was mapped to the hg38 human reference genome, and the genomic positions of the public GWAS hg19 results were converted to hg38 using the liftOver R package [43]. We then calculated PGS for each pain phenotype by using PRSice 2.0 [44]. To minimize the number of comparisons and to obtain the full cumulative effects of genome-wide SNPs, a fixed *p*-value threshold was kept at 1.0 (P_T_ = 1). Default pruning parameters were used. All PGSs for each chronic pain were standardized. To ensure our results were not specific to the PRSice-2 tool, we additionally used another method, PRS-CS, a state-of-the-art approach estimating polygenic scores via Bayesian regression. Since the Bayesian approach of PRS-CS with small sample size showed reduced performance, we here primarily employed the PRSice 2.0 method, then further confirmed the results by using PRS-CS [45].

### Statistical analysis

Generalized logistic regression models of case-control status (binary) on PGS (continuous). Unless otherwise specified, statistical analyses included sex and ancestry principal components (PCs, PC1-PC5) as covariates to account for potential residual effects of population stratification and cryptic relatedness. In models, any specific PCs were excluded as covariates when effective convergence failed due to separation. Firth’s bias-reduced logistic regression, which is effective in handling complete separation in standard logistic regression [46], was additionally used to address any failed models, confirming the model including all of PC1-PC5 yielded consistent results.

The initial assessment involved examining 1) the association of continuous pain PGSs with each chronic pain clinical diagnosis (binary y/n), and 2) the association of chronic pain PGSs with suicide death case-control status. Follow-up models examined effects in males and females separately. Follow-up tests also examined the PGS-suicide death associations stratified by presence/absence of chronic pain diagnosis to evaluate whether the chronic pain diagnosis moderated PGS-suicide death associations.

We also compared the prevalence of suicide death among groups categorized by PGS quartiles to evaluate whether individuals in the top quartile are disproportionately represented in the case group, in order to assess the potential future clinical utility of the two phenotypes and the effects of categorical stratification, following the approach used in previous PGS studies [47–53]. To test this, we partitioned the cohort into three groups: 1) low PGS group in 1^st^ quartile of PGS, 2) intermediate PGS within the 2^nd^ and 3^rd^ quartiles, and 3) high PGS within the 4^th^ quartiles. Comparisons of the defined groups were then conducted using generalized logistic regression models adjusting sex and PC1-PC5 for covariates.

### Pathway-based PGS analysis

Pathway-based PGSs were calculated by incorporating the effects of only variants within a gene pathway. To identify putative gene sets, an enrichment analysis with gene ontology pathways was performed by using ConsensusPathDB [54]. This analysis included genes (promoter and gene body) encompassing SNPs at a significant level (*p*-value < 0.005) within each of the chronic pain GWAS. The pathway-based PGSs within enriched pathways were then calculated using PRSSet [55], a module of PRSice-2 with consistent parameters used for estimating genome-wide PGS described above. Subsequently, the pathway-based PGS were examined for associations with suicide death using generalized logistic regression models and adjusting for sex and PC1-PC5 as covariates.

### PGS association analysis of extended other specific pain types

In consultation with a chronic pain specialist (A.O.), we expanded our analysis to include five specific types of pain diagnoses: monoarticular arthritis [56], back pain [57], chronic inflammatory demyelinating polyneuropathy [56], irritable bowel syndrome [58], and knee pain [59], Information regarding these traits in the context of pain is detailed in the Supplementary Methods. GWAS for monoarticular arthritis and chronic inflammatory demyelinating polyneuropathy were conducted in 456,348 individuals. GWAS for back pain, irritable bowel syndrome, and knee pain included 453,862, 486,601, and 171,516 individuals, respectively. All participants were from the UK Biobank European cohort. This extension aimed to evaluate an array of specific pain phenotypes that may share a genetic relationship with suicide death. Publicly available summary statistics results from the largest available GWAS were used to calculate the relevant PGS using the methods outlined above. Additionally, a multiple logistic regression (multivariate) test was conducted on PGSs for all types of chronic pain to assess specificity of associations of types of pain with suicide death.

### Shared genetic covariation among pain phenotypes

We estimated the pair-wise cross-trait genetic correlations across all analyzed pain types using linkage disequilibrium score regression (LDSC) to assess genetic overlap Summary statistic data were utilized to calculate molecular genetic correlations (*r*_*g*_) based on the common SNPs from the 1000 Genomes European ancestral data by using the LDSC python package with default parameters. The significance threshold was defined at a Bonferroni corrected p-value < 0.05. In addition, suicide death cases were clustered based on the PGSs associated significantly with chronic pain types using the principal component analysis approach, while adjusting for sex and PC1-PC5 as covariates.

### Association analysis between PGSs and suicide death, controlling for psychiatric conditions and sleep disorders

Given the likelihood of increased prevalence of psychiatric conditions among suicide death cases with chronic pain (Supplementary Table S2), we sequentially conducted association tests of PGSs with suicide death excluding all individuals with specific psychiatric diagnoses: substance use disorders, anxiety, bipolar disorder, major depressive disorder, opioid use disorders, and sleep-related disorders. The aim of this analysis was to evaluate whether chronic pain is independently associated with suicide risk.

### Mendelian randomization analysis

To investigate the potential causal relationship between chronic pain and suicide death, we performed two-sample Mendelian randomization (MR) analyses from each chronic pain type to suicide death using the *MendelianRandomization* R package [60]. Instrumental variables (IVs) were selected according to three core assumptions: (1) IVs are significantly associated with the exposure (i.e., each chronic pain type), (2) IVs are not directly associated with the outcome (suicide death), and (3) IVs are not associated with potential confounders. We first identified genome-wide significant SNPs (*p*-value < 5e-8) from pain GWAS summary statistics and applied linkage disequilibrium clumping (r2 < 0.001, 1000 kb; European 1000 Genomes reference) to select independent lead SNPs. We then removed SNPs associated with potential confounders, including alcohol use disorders, body mass index, smoking, neuroticism, and sleep disorders, identified from GWAS catalog [61]. To avoid weak instrument bias, we calculated *F*-statistics and excluded IVs with F < 10. Causal inference was evaluated using the inverse variance weighted method, with MR-Egger [62] and MR-PRESSO [63] applied to assess the potential bias of directional and horizontal pleiotropy, respectively.

## Results

The detailed summary of the sample cohort analyzed in this study has been previously described [35]. Basic demographic information of suicide death cases with (SD-CP) and without (SD-NCP) chronic pain is presented in Supplementary Table S2.

### Polygenic scores for chronic pain reflect the prevalence of clinically diagnosed chronic pain

Of the suicide death cases, 170 (17.24%) had a chronic pain diagnostic history. PGS_MCP_ and PGS_CWP_ were both significantly elevated in cases with chronic pain compared to those without chronic pain: OR = 1.40 (CI = 1.18–1.65) and 1.19 (CI = 1.01–1.41); *p*-value = 1.01e-4 and 0.034, respectively (Supplementary Fig. S1).

### Association of Chronic Pain PGS with suicide death risk

**Fig. 2:**
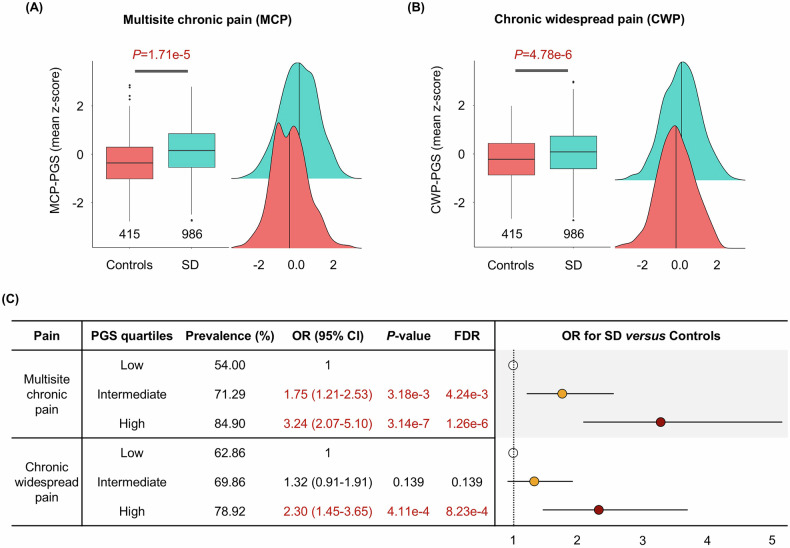
Association between PGS for chronic pain (CP) types and suicide death risk. **A-C** Boxplots with distributions of PGS_MCP_ (**A**) and PGS_CWP_ (**B**) between non-suicide deaths and suicide deaths. X-axis and Y-axis represent control/case and the PGSs for CP, respectively. **C** Comparisons results including *p*-value and odds ratio of SD versus control according to the three risk partitions of PGS_MCP_ and PGS_CWP_, respectively, (low vs intermediate and low vs high): Low: low PGS group (PGSs for CP in the first quartile), Intermediate: intermediate PGS group (PGSs in the second and third quartiles), and High: high PGS group (PGSs in the fourth quartile). Control: non-suicide general control population; SD: postmortem suicide death cases.

PGS_MCP_ and PGS_CWP_, considered as continuous variables, were both significantly associated with suicide death: PGS_MCP_ OR = 1.40 (CI = 1.20–1.64); *p*-value = 1.71e-5 (FDR = 1.71e-5) and PGS_CWP_ OR = 1.45 (CI = 1.24–1.70); *p*-value = 4.78e-6 (FDR = 9.56e-6) (Fig. 2A and B). Moreover, consistent results were observed within PGS-stratified groups (Fig. 2C).

### PGS across Controls, SD-NCP, and SD-CP

**Fig. 3:**
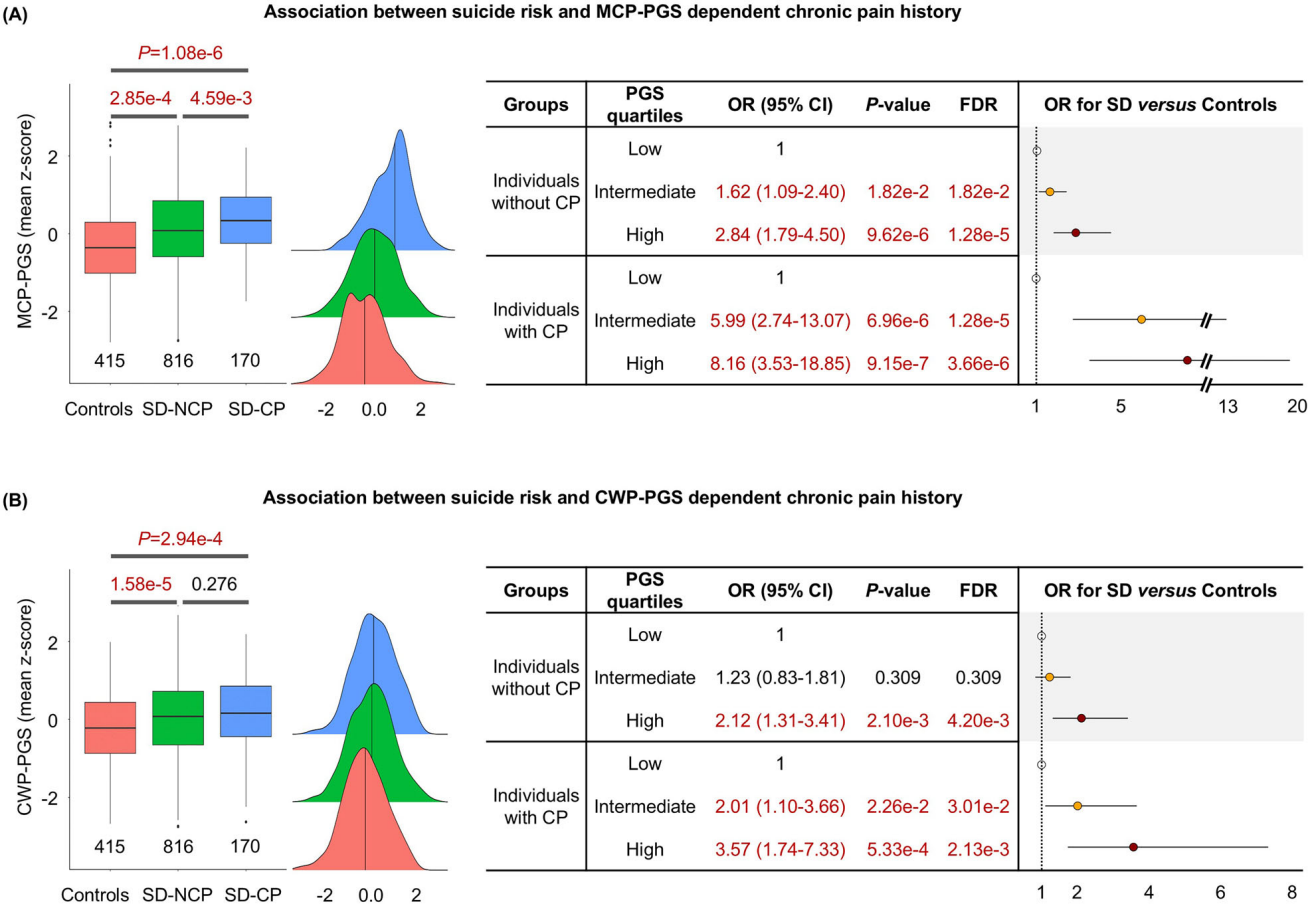
Comparisons across three subgroups: control, suicide death without chronic pain, and suicide death with chronic pain. **A** Association between suicide risk and PGS_MCP_ dependent on chronic pain history. The left panel shows boxplots with distributions of PGS_MCP_ in control, SD-NCP (suicide death without chronic pain), and SD-CP (suicide death with chronic pain). X-axis and Y-axis represent subgroups and the PGS_MCP_, respectively. The middle panel depicts density plots of PGS_MCP_ for control, SD-NCP, and SD-CP, respectively. The right panel represents comparisons results including *p*-value and odds ratio of SD versus control according to the three risk partitions of PGS_MCP_ in individuals without and with CP. **B** Association between suicide risk and PGS_CWP_ dependent chronic pain history. All attributes for (**B**) are consistent with (**A**). Control: non-suicide general control population; SD-NCP: postmortem suicide death cases without chronic pain; SD-CP: postmortem suicide death cases with chronic pain; MCP: multisite chronic pain; CWP: chronic widespread pain.

To evaluate if clinical chronic pain diagnosis influences the association between PGS and suicide death risk, we compared PGS_MCP_ and PGS_CWP_ across three subgroups: controls, SD-NCP (e.g., suicide death without chronic pain), and SD-CP (e.g., suicide death with chronic pain). The levels of PGS_MCP_ were significantly elevated in both SD-NCP (OR = 1.34; CI = 1.14–1.57; *p*-value = 2.85e-4) and SD-CP (OR = 1.84; CI = 1.44–2.35; *p*-value = 1.08e-6) as compared with controls (Fig. 3A). Similarly, PGS_CWP_ demonstrated a significant increase in both SD-NCP (OR = 1.44; CI = 1.22–1.70; *p*-value = 1.58e-5) and SD-CP (OR = 1.55; CI = 1.22–1.97; *p*-value = 2.94e-4) than in controls (Fig. 3B). Moreover, in quartile stratified models, individuals in the highest PGS groups of both PGS_MCP_ and PGS_CWP_ exhibited a higher prevalence of suicide death compared to those in the lowest quartile regardless of whether they had a history of chronic pain diagnosis (right panels in Fig. 3). This suggests that genetic signals associated with PGS_MCP_ and PGS_CWP_ may be linked to the risk of suicide death irrespective of chronic pain diagnostic history in EHR, though it is noteworthy that the ORs for suicide death prevalence in both MCP and CWP were amplified in individuals with a history of chronic pain. All significant results reflected FDR < 0.05.

### Assessment of sex-related effect on PGS associations

Sex-stratified analyses found the associations of PGS_MCP_ (Supplementary Figs. S2A and S2B) and PGS_CWP_ (Supplementary Figs. S3A and S3B) with suicide death status were significant in both males and females. When comparing between highest and lowest quartile of PGSs for MCP and CWP, individuals in the highest PGS quartile showed a significantly higher prevalence of suicide death (Supplementary Figs. S2C and S3C).

### Pathway-based PGS association analysis

We did not find pathway-based PGSs for MCP and CWP within potential gene sets significantly associated with suicide risk.

### PGS association analysis with extended specific pain types

**Fig. 4:**
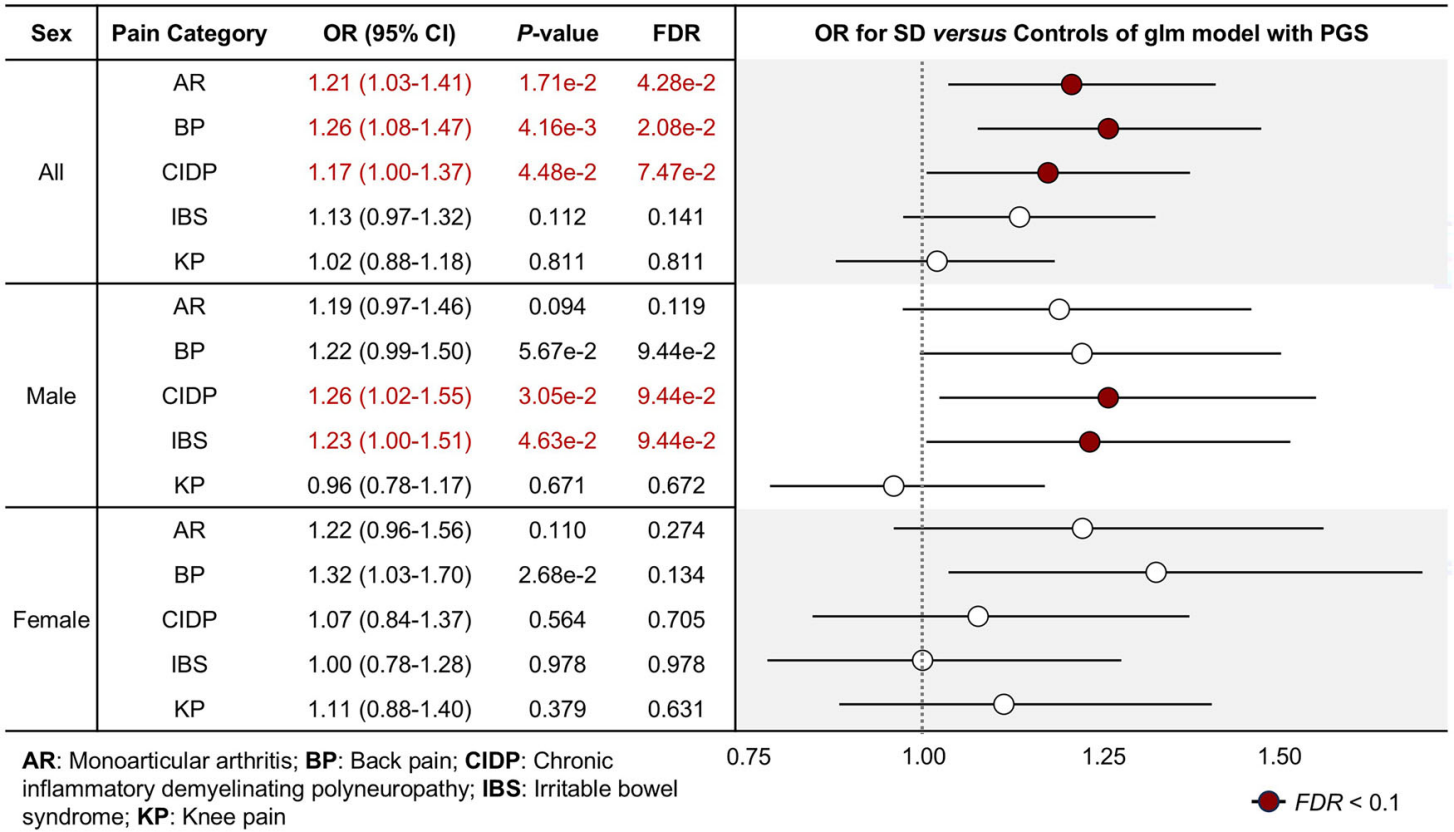
The association of PGS as a continuous variable for extended different pain types and suicide risk using generalized logistic regression model, adjusting for sex and principal components (PCs; PC1-PC5). The results include odds ratios from the model between status of case/control and PGS in all (top), male (middle), and female (bottom).

Given the significant associations with global pain phenotypes, we expanded our PGS association analysis to include five additional specific pain conditions: monoarticular arthritis, back pain, chronic inflammatory demyelinating polyneuropathy, irritable bowel syndrome, and knee pain. Among these, three of the five (PGS_monoarticular arthritis_; OR = 1.21, PGS_back pain_; OR = 1.26, and PGS_chronic inflammatory demyelinating polyneuropathy_; OR = 1.17) were significantly associated with suicide death (Fig. 4). Sex-stratified association analyses identified one additional suicide death-associated PGS (PGS_irritable bowel syndrome_; OR = 1.23) in males only. That is, PGSs for four additional pain types were found to be increased in suicide death compared with controls, considering sex information. Analysis of the PGS quartiles were consistent across three of the pain subtypes (monoarticular arthritis, back pain, and chronic inflammatory demyelinating polyneuropathy) (Supplementary Fig. S4).

### Genetic relationships across pain types

**Fig. 5:**
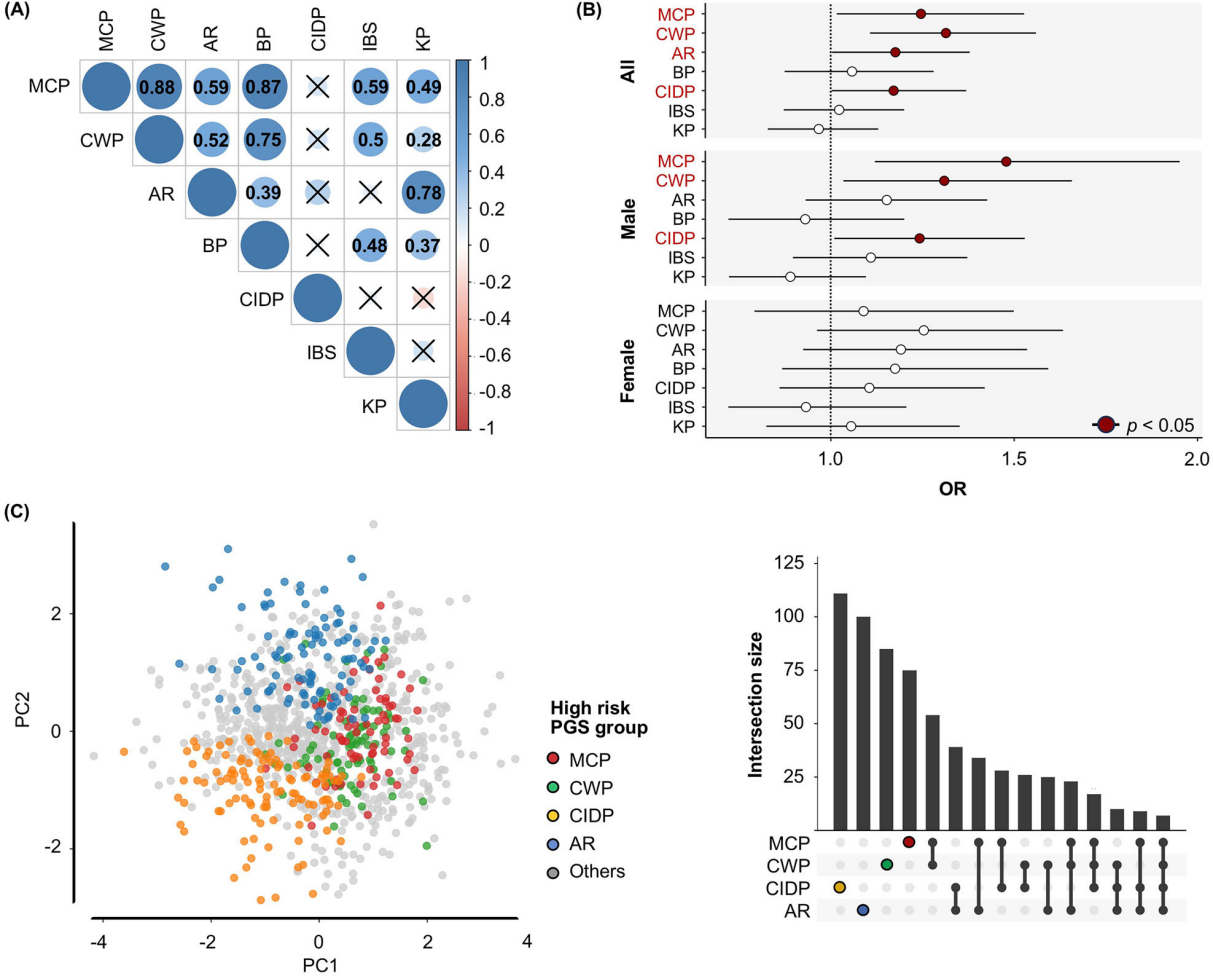
Genetic correlations across the seven types of chronic pain and multivariate association test between chronic pain (CP) and suicide death (SD) risk. **A** The pairwise genetic correlations across seven studied pain types through linkage disequilibrium score regression (LDSC) analysis. The X symbol represents insignificance. **B** A multivariate association test between PGSs for the seven CP types and SD risk, stratified by sex. X-axis and Y-axis represent odds ratio adjusted by covariates (including sex and principal components PC1-PC4) and CP types, respectively. **C** Clustering SD cases based on four PGSs for MCP, CWP, CIDP, and AR by utilizing principal component analysis approach. SD cases within exclusively high PGS group were defined by posing within 4^th^ quartile of PGS for each of four CP types. The right panel represents the numbers of SD cases intersected in high PGS groups among four CP types. The first four bars indicate exclusively high PGS groups for each CP type: MCP, CWP, CIDP, and AR, depicted in red, green, yellow, and blue, respectively. MCP: Multisite chronic pain; CWP: Chronic widespread pain; AR: Monoarticular arthritis; BP: Back pain; CIDP: Chronic inflammatory demyelinating polyneuropathy; IBS: Irritable bowel syndrome; KP: Knee pain.

LDSC analyses observed high genetic correlations among several types of chronic pain including MCP, CWP, and back pain, suggesting that a common genetic mechanism among them could contribute to the relationship with suicide death risk. Interestingly, chronic inflammatory demyelinating polyneuropathy was not significantly genetically correlated with any of the other pain conditions (Fig. 5A), despite still being associated with suicide death risk (Fig. 4).

Since genetic correlations were observed across different types of chronic pain, a multivariate association test was conducted between PGSs for all pain types and suicide death risk. Four chronic pain types, including MCP, CWP, monoarticular arthritis, and chronic inflammatory demyelinating polyneuropathy, remained significantly associated with suicide death risk (Fig. 5B). In this multivariate analysis, principal component 5 (PC5) was excluded as a covariate due to convergence issues and evidence of complete separation, and confirmed that the Firth’s bias-reduced logistic regression model including PC1-PC5 (Supplementary Table S3) observed results consistent with those obtained using standard logistic regression with PC1-PC4.

Furthermore, utilizing principal component analysis clustering analysis involving PGSs for the four pain types, sex, and PCs, suicide death cases were classified into subgroups based on exclusively highest quartile PGS groups within each specific type of chronic pain (Fig. 5C). As expected, suicides within high PGS risks for MCP and CWP highly overlapped, while monoarticular arthritis and chronic inflammatory demyelinating polyneuropathy are more distinct.

Mendelian randomization found no evidence for a causal relationship between any chronic pain type and suicide death (data not shown).

### Association analysis controlling for psychiatric conditions

In the case of MCP and CWP, associations remained significant after controlling for five psychiatric conditions and sleep related disorders (Supplementary Table S4). However, PGS_chronic inflammatory demyelinating polyneuropathy_ lost significance when controlling for anxiety and major depressive disorder. Furthermore, the associations of PGS_monoarticular arthritis_ with suicide death were not significant after controlling for sleep-related disorders. These suggest that psychiatric phenotypes might be moderating some of the observed effects.

### Analysis of PGS calculated by PRS-CS

In the association tests for each pain type by using PRS-CS, all of results were consistently observed to be significantly associated with suicide (Supplementary Fig. S5A). The multivariate association analysis also observed consistent results; only monoarticular arthritis was not significant, however the association remained positive (odds ratio=1.145) and marginal (*p*-value = 0.095), suggesting that a larger sample size may be required to confirm this finding (Supplementary Fig. S5B).

## Discussion

In this study, we analyzed whole-genome sequencing and diagnostic data from population-based suicide deaths in Utah incorporating various chronic pain GWAS summary-based statistical results. Our analysis provides a detailed characterization of the genetic architecture underlying chronic pain types, and specifically explored the connection to risk of suicide death. We observed polygenic overlaps of several chronic pain types with suicide death risk. These results support findings of a prior twin study, which suggested that shared molecular mechanisms (e.g., shared genetic etiology) rather than a physiologic effect of a pain condition itself, may contribute to the co-occurrence of these phenomena [21]. Our results showed potential further evidence that the clinical associations between types of pain and suicide death could be, in part, attributable to shared genetic risks.

The shared genetic risk between chronic pain and suicide death was not dependent upon clinical diagnosis of chronic pain. In particular, significant genetic associations between suicide death and PGS_MCP_ and PGS_CWP_, were retained irrespective of the presence of a chronic pain diagnosis. This implies that the suicide death, MCP, and CWP genetic predispositions overlap and are not solely influenced by exposures associated with chronic pain, as consistently observed in a previous co-twin study [21]. As such, it appears that treating chronic pain may not fully eliminate the underlying risk of suicide death. Therefore, genetic risk models identifying a subset of suicide death linked to chronic pain could significantly help understand this aspect of suicide risk, especially for individuals who have not yet been clinically diagnosed with chronic pain or who are poorly detected in healthcare settings. However, presence of clinical chronic pain was associated with additional risk for suicide death. Thus, both shared genetic liability and clinical presence of chronic pain likely synergistically contribute to the risk of suicide death, suggesting that EHR-integrated genetic models could enhance detection and potentially improve preventative risk models.

One potential shared mechanism is sensitivity in neural systems. Previous studies have observed that sensitivity to physical pain overlaps with sensitivity to emotional pain, such as social rejection [64, 65]. Additionally, other shared physiological processes including sleep regulation and pain signal processing, especially development of hyperalgesia, may also serve as potential mechanisms [66]. The characterization of polygenic associations will allow future mechanistic analyses using the functional variation in this whole-genome sequencing data resource.

Another distinct feature of our study is the assessment of polygenic risk associated with a broad range of chronic pain types in relation to suicidality. A significant challenge in exploring the clinical impact of chronic pain conditions on suicide is the high correlations among various chronic pain types, resulting in their co-occurrence [67, 68]. The genetic assessments in this study provide an insight into a comprehensive view of the genetic relationships between different chronic pain types and overlap with risk of suicide death.

We performed a control analysis with a putatively unrelated phenotype, hair color. No significant association between suicide death risk and the control PGS for hair color was identified as depicted in the Supplementary Methods and Supplementary Fig. S6.

CWP is also strongly clinically and genetically associated with MCP [41]. In our analysis, PGS_MCP_ could be significantly used to predict CWP, showing a high genetic correlation (*r*_g_ = 0.88), consistent with published results [41]. Therefore, there may not be a clinical cutoff point distinguishing these two pain types [69, 70]. However, some evidence suggests that CWP may not simply be an extension of MCP, as localized pain in multisite conditions and the manifestation of pain throughout the entire body (CWP) may exhibit considerably different clinical phenotypes [41, 71]. In our study, PGS_MCP_ and PGS_CWP_ were derived from two separate GWASs, each involving clinically distinct sample sets [41]. Our multivariate analysis showed that both MCP and CWP were significantly and independently associated with suicide death risk, suggesting unique aspects of MCP and CWP genetic architectures contributing differently to the risk of suicide death.

Chronic inflammatory demyelinating polyneuropathy demonstrated distinct associations in our analyses. A prevalent symptom of chronic inflammatory demyelinating polyneuropathy is neuropathic pain involving heightened responsiveness in the brain to stimuli (hyperalgesia) [72, 73]. Patients with polyneuropathy have demonstrated 2.3 times greater suicide prevalence than the general population [74]. Our results indicated overlapping genetic factors in chronic inflammatory demyelinating polyneuropathy and suicide death, suggesting a contribution to their comorbidity. Specifically, chronic inflammatory demyelinating polyneuropathy exhibited genetic differentiation from other pain conditions and showed significance in the multivariate association test. Our findings provide new insights into a unique pattern of genetic overlap of chronic inflammatory demyelinating polyneuropathy and suicide death not shared with other pain conditions. This insight may help identify chronic inflammatory demyelinating polyneuropathy-specific mechanisms and facilitate the identification of a distinct genetic subgroup of individuals at risk for suicide death [75–78].

In addition to analysis of PGS as a continuous variable, we compared sub-categorized risk groups based on quartiles of PGS to determine whether individuals within the upper quartile PGS group exhibit an increased risk of suicide death compared to the lower quartile group as a baseline. This categorical approach is commonly used to assess the potential utility of PGS as a tool for stratifying high PGS individuals. Our results mirrored qualitative associations, suggesting potential future utility of this approach [47, 49–53].

One previous study, using 2-sample bidirectional MR, observed a causal relationship of multisite chronic pain to suicide death [79], while Our unidirectional MR analysis did not identify significant results of any pain types; however, future analyses of a larger SD cohort are expected to inform these relationships.

Given the significant genetic and phenotypic correlations across general chronic pain types and suicide death, it is possible that a genetic factor score using genomic structural equation modeling will account for significantly more variation in suicide death than PGS from single chronic pain discovery GWAS in future studies.

We acknowledge several general limitations in this study. Firstly, the suicide death cohort included a relatively small number of females due to the increased general prevalence of suicide death in males. The observed associations, particularly in a sex-dependent manner, might be attributed to underpowered statistical analyses in females. Additionally, the number of local ancestry-matched control samples in the general population was small relative to the number of suicide death samples. Replication studies with larger samples of females and controls are warranted. It is important also to note that this study was limited to individuals of European ancestral admixtures due to data availability. Also, these analyses may be less well-powered given modest discovery GWAS sample sizes for several specific pain phenotypes, but nevertheless suggest significant associations with specific pain subtype. Finally, future studies will need to incorporate more comprehensive demographic and clinical information to dissect other factors, including additional clinical conditions, that may influence the relationships between chronic pain and the risk of suicide death.

In conclusion, our integrative study, utilizing genomic and EHR data, examined the genetic pathways associated with various pain types that overlap with the risk of suicide death. These findings have the potential to advance our understanding of clinical comorbidities through a genetic etiological lens, indicating possible utility in classifying a subgroup of suicide deaths with high genetic and phenotypic liability for chronic pain. Our results offer valuable insights for future clinical studies, providing a supportive tool for the development of future prevention and intervention strategies.

## Supplementary information

Supplementary Figures

Supplementary Tables

Supplementary Methods

## Data Availability

Publicly available GWAS datasets investigated in this study are available from the following sources. Multisite chronic pain: https://www.ebi.ac.uk/gwas/publications/31194737. Chronic widespread pain: https://zenodo.org/records/4459546. Monoarticular arthritis: https://www.ebi.ac.uk/gwas/publications/34737426. Back pain: https://zenodo.org/records/1319332, Chronic inflammatory demyelinating polyneuropathy: https://www.ebi.ac.uk/gwas/publications/34737426. Irritable bowel syndrome: https://www.ebi.ac.uk/gwas/publications/34741163, and Knee pain: https://www.ebi.ac.uk/gwas/publications/31482140. Additional data from this study is available from the authors upon request.

## Code availability

PRSice-2: https://choishingwan.github.io/PRSice/; PRS-CS: https://github.com/getian107/PRScs; LDSC: https://github.com/bulik/ldsc; PLINK v1.9: https://www.cog-genomics.org/plink/1.9.
