# Supplementary material for: Genetic risk of chronic pain conditions associated with risk of suicide death through an integrative analysis of EHR and genomics data
Source: Transl Psychiatry. 2026 Feb 16;16:117. doi: 10.1038/s41398-026-03861-6 (PMC12949045; doi:10.1038/s41398-026-03861-6)
Supplement: Supplementary file 1 — Supplementary Figures [file 41398_2026_3861_MOESM1_ESM.pdf]

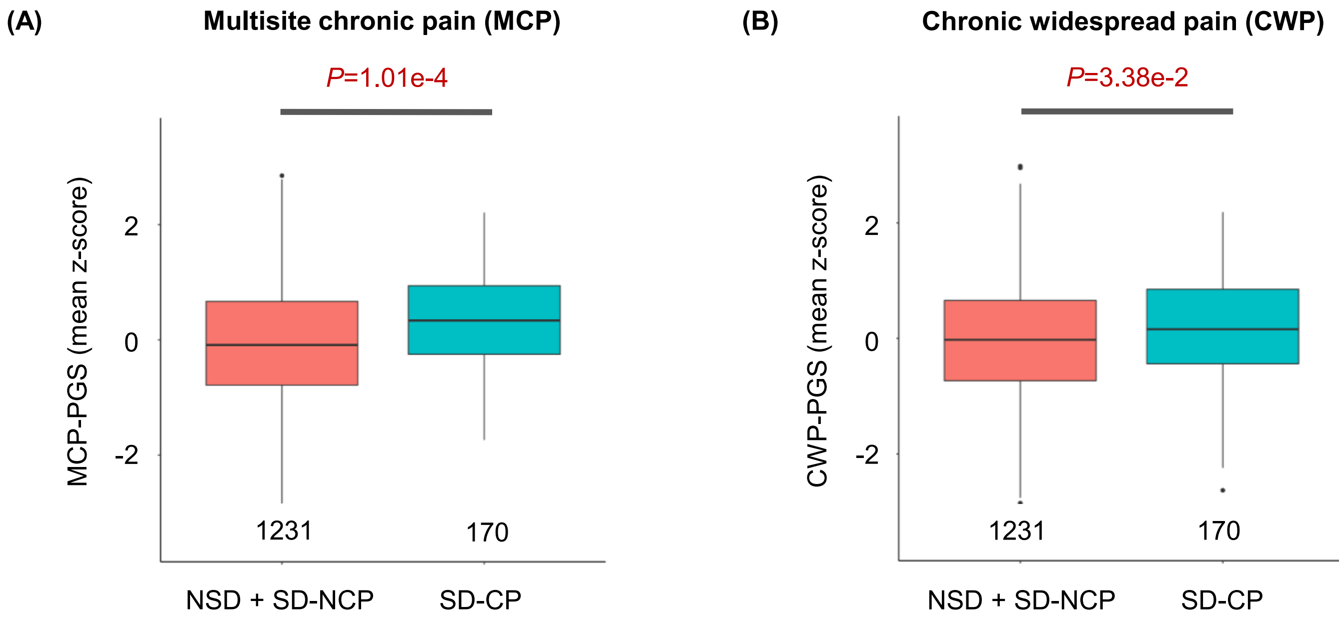

Supplementary Figure S1. The prevalence of clinical chronic pain (CP) diagnoses associated with polygenic scores for CP. Boxplots with distribution of PGS for multisite chronic pain (A) and chronic widespread pain (B) between individuals with and without CP. X-axis and Y-axis represent individuals with and without CP and the PGSs for CP, respectively. Controls (NSD): non-suicide general control population; SD-NCP: postmortem suicide death cases without chronic pain; SD-CP: postmortem suicide death cases with chronic pain.

Association between suicide risk and MCP-PGS stratified by sex

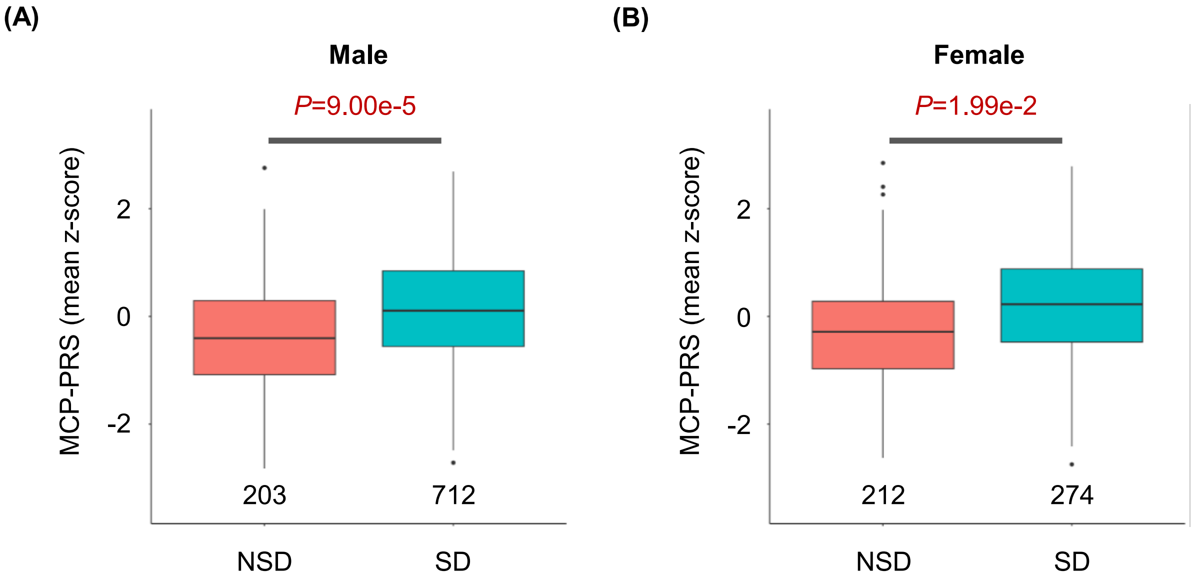

(C)

Sex	PGS quartiles	Prevalence (%)	OR (95% CI)	*P*
M	Low	63.32	1	
	Intermediate	80.31	1.92 (1.18-3.13)	8.90e-3
	High	87.34	3.30 (1.79-6.09)	1.35e-4
F	Low	38.52	1	
	Intermediate	54.13	1.47 (0.82-2.64)	0.193
	High	78.69	3.34 (1.68-6.64)	5.73e-4

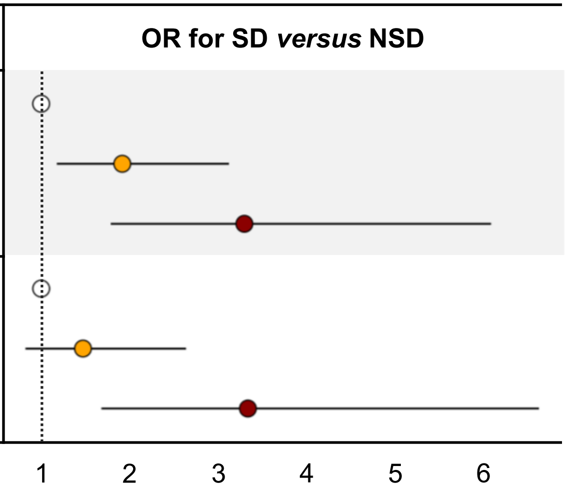

Supplementary Figure S2. Association between SD risk and PGS for MCP stratified by sex. (A, B) Boxplots with distributions of PGS_{MCP} between NSD and SD in male (A) and female (B). (C) Comparisons results including p-value and odds ratio of SD versus NSD according to the three PGS partitions of PGS_{MCP} in male and female. NSD: non-suicide general control population; SD: postmortem suicide death cases; MCP: multisite chronic pain.

Association between suicide risk and CWP-PRS stratified by sex

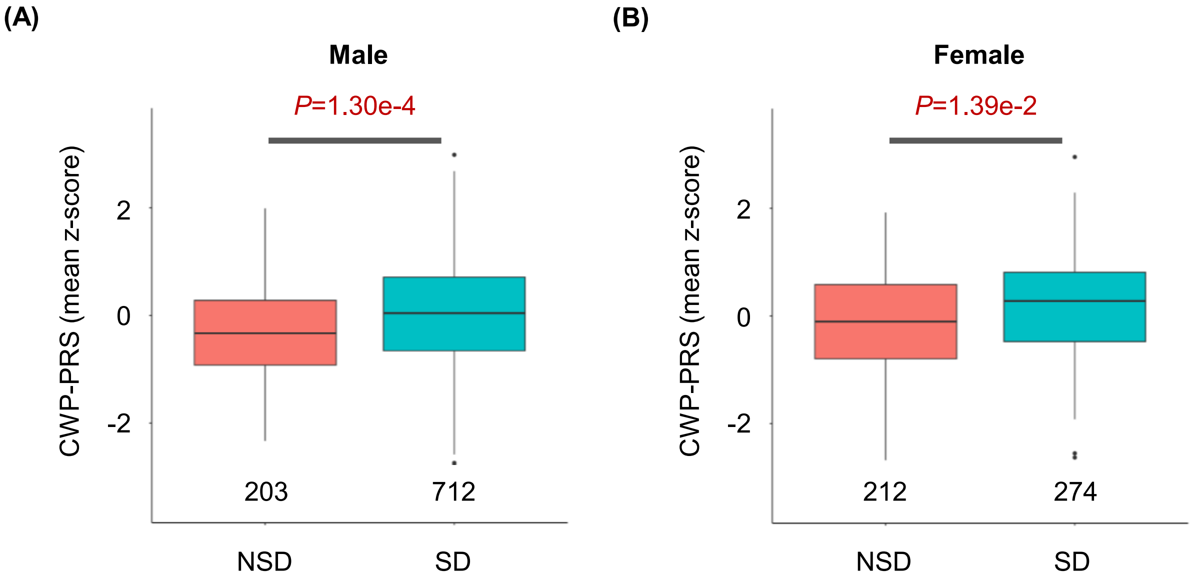

(C)

Sex	PGS quartiles	Prevalence (%)	OR (95% CI)	*P*
M	Low	69.87	1	
	Intermediate	77.24	1.47 (0.89-2.41)	0.130
	High	86.90	2.67 (1.44-4.96)	1.87e-3
F	Low	47.54	1	
	Intermediate	57.02	1.28 (0.72-2.25)	0.398
	High	63.93	1.84 (0.92-3.69)	8.60e-2

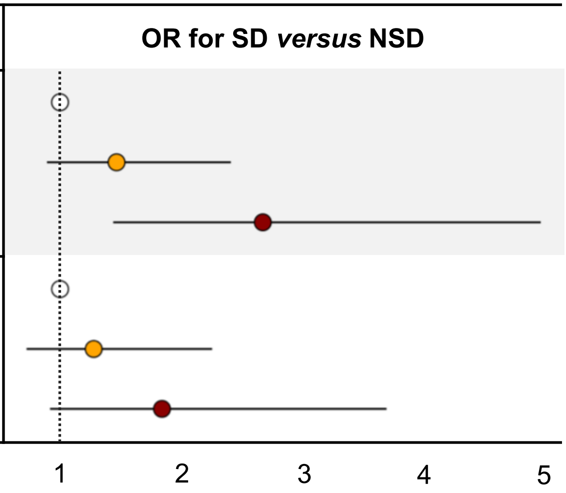

Supplementary Figure S3. Association between SD risk and PGS for CWP stratified by sex. (A, B) Boxplots with distributions of PGS_{CWP} between NSD and SD in male (A) and female (B). (C) Comparisons results including p-value and odds ratio of SD versus NSD according to the three PGS partitions of PGS_{CWP} in male and female. NSD: non-suicide general control population; SD: postmortem suicide death cases; CWP: chronic widespread pain.

Sex	Pain Category	OR (95% CI)	*P*-value	*FDR*
All	AR	1.83 (1.20-2.81)	5.42e-3	1.89e-2
	BP	1.84 (1.18-2.88)	7.57e-3	1.89e-2
	CIDP	1.82 (1.14-2.89)	1.17e-2	1.96e-2
	IBS	1.27 (0.81-2.00)	0.299	0.373
	KP	0.90 (0.59-1.39)	0.634	0.634
Male	AR	1.51 (0.85-2.65)	0.157	0.196
	BP	1.73 (0.97-3.09)	0.062	0.156
	CIDP	2.38 (1.27-4.49)	7.14e-3	3.57e-2
	IBS	1.62 (0.87-3.00)	0.127	0.196
	KP	0.81 (0.46-1.45)	0.482	0.482
Female	AR	2.44 (1.23-4.82)	1.04e-2	5.19e-2
	BP	1.82 (0.88-3.77)	0.105	0.263
	CIDP	1.36 (0.67-2.76)	0.40	0.666
	IBS	1.04 (0.51-2.12)	0.916	0.916
	KP	1.11 (0.57-2.17)	0.761	0.916

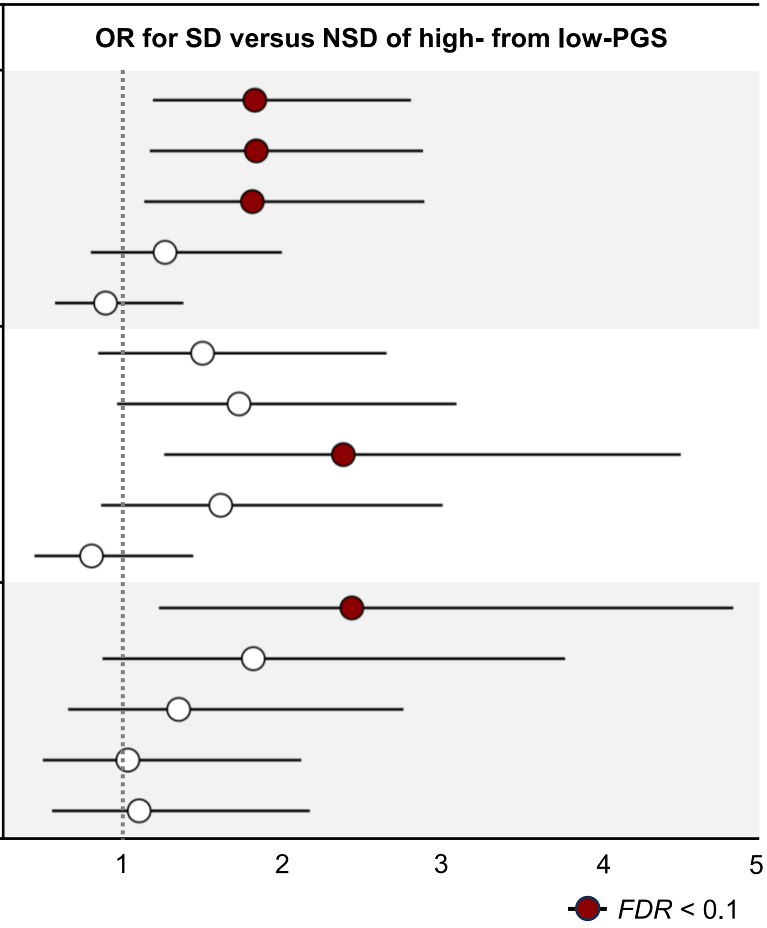

AR: Monoarticular arthritis; **BP**: Back pain; **CIDP**: Chronic inflammatory demyelinating polyneuropathy; **IBS**: Irritable bowel syndrome; **KP**: Knee pain

Supplementary Figure S4. The association of PGS for extended different pain types and suicide risk. The results include odds ratios of SD prevalence in high-PGS (4th quartile) groups of five extended types of pain compared to low-PGS (1st quartile) groups serving as baselines in all (top), male (middle), and female (bottom).

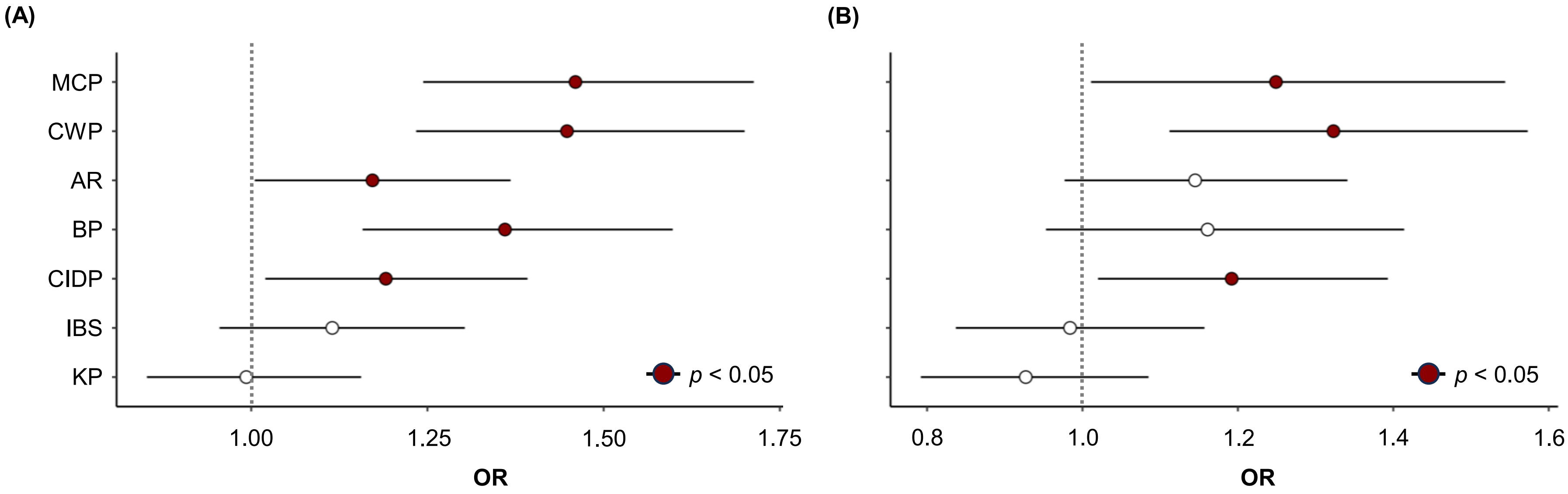

Supplementary Figure S5. The association analysis of PGS calculated by using PRScs. (A) An association analysis results by performing a logistic regression for each individual pain type, and (B) a multivariate association analysis for the chronic pain types. MCP: Multisite chronic pain; CWP: Chronic widespread pain; AR: Monoarticular arthritis; BP: Back pain; CIDP: Chronic inflammatory demyelinating polyneuropathy; IBS: Irritable bowel syndrome; KP: Knee pain.

Sex	Hair colors	OR (95% CI)	*P*-value
All	Hair 1	0.92 (0.79-1.07)	0.280
	Hair 2	1.01 (0.87-1.18)	0.871
	Hair 3	0.91 (0.78-1.06)	0.238
	Hair 4	0.97 (0.84-1.13)	0.717
Male	Hair 1	0.91 (0.74-1.12)	0.371
	Hair 2	0.98 (0.80-1.20)	0.835
	Hair 3	0.94 (0.77-1.15)	0.571
	Hair 4	1.02 (0.83-1.25)	0.855
Female	Hair 1	0.89 (0.70-1.15)	0.390
	Hair 2	1.03 (0.81-1.31)	0.817
	Hair 3	0.92 (0.72-1.17)	0.491
	Hair 4	0.88 (0.69-1.12)	0.304

OR for SD *versus* NSD of high- from low-PRS

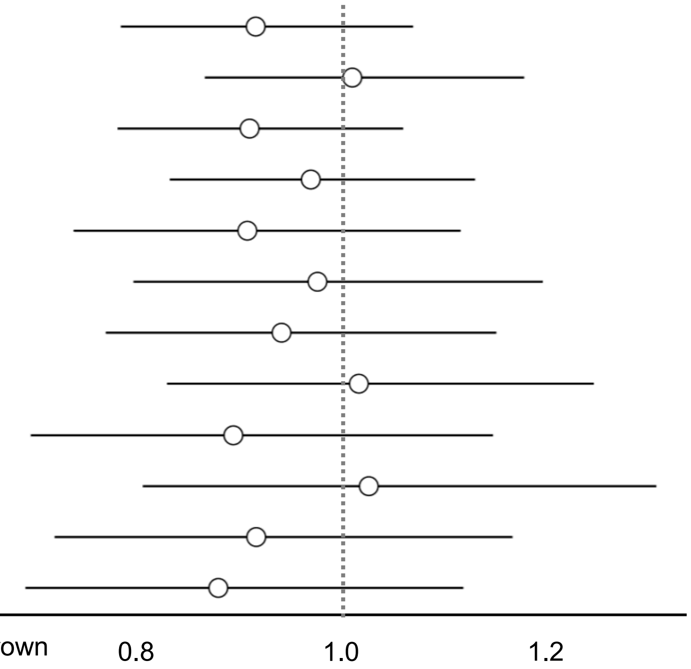

Hair 1: Light brown; **Hair 2**: Black; **Hair 3**: Red; **Hair 4**: Dark brown

Supplementary Figure S6. Association test between SD risk and PGSs for hair colors, stratified by sex.
