# Supplementary material for: Genetic risk of chronic pain conditions associated with risk of suicide death through an integrative analysis of EHR and genomics data
Source: Transl Psychiatry. 2026 Feb 16;16:117. doi: 10.1038/s41398-026-03861-6 (PMC12949045; doi:10.1038/s41398-026-03861-6)
Supplement: Supplementary file 2 — Supplementary Tables [file 41398_2026_3861_MOESM2_ESM.pdf]

Supplementary Table S1. ICD codes for chronic pain phecode (338.3) defining cases with chronic pain phenotypes

ICD Version	ICD Code	Code Description
ICD9	338.2	Chronic pain
ICD9	338.21	Chronic pain due to trauma
ICD9	338.22	Chronic post-thoracotomy pain
ICD9	338.28	Other chronic postoperative pain
ICD9	338.29	Other chronic pain
ICD10	G89.2	Chronic pain, not elsewhere classified
ICD10	G89.21	Chronic pain due to trauma
ICD10	G89.22	Chronic post-thoracotomy pain
ICD10	G89.28	Other chronic postprocedural pain
ICD10	G89.29	Other chronic pain
ICD10	G89.4	Chronic pain syndrome

Supplementary Table S2. Basic demographic information of suicide death cases with and without chronic pain

	Suicide deaths	
	**SD[a] without CP[b]**	**SD with CP**
N	816	170
Female, n (%)	207 (25.37%)	67 (39.41%)
Psychiatric characteristics, n (%)		
Substance use disorders	245 (30.02%)	124 (72.94%)
Anxiety	345 (42.28%)	148 (87.06%)
Bipolar disorder	287 (35.17%)	109 (64.12%)
Major depressive disorder	258 (31.62%)	108 (63.53%)
opioid use disorders	91 (11.15%)	80 (47.06%)
Schizophrenia	41 (5.02%)	18 (10.59%)
Sleep related disorders	165 (20.22%)	111 (65.29%)

a. Suicide death
b. Chronic pain

Supplementary Table S3. Multivariate analysis using Firth's bias-reduced logistic regression

Pain type	PRSice-2[a]		PRS-CS[b]	
	OR[c]	p-value	OR[c]	p-value
Multisite chronic pain	1.504	0.01551731	1.249	0.0397299
Chronic widespread pain	1.469	0.01032513	1.323	0.00157135
Monoarticular arthritis	1.352	0.03049319	1.307	0.06998335
Back pain	1.237	0.70669549	1.356	0.142254
Chronic inflammatory demyelinating polyneuropathy	1.348	0.03512326	1.356	0.0226104
Irritable bowel syndrome	1.113	0.70096948	1.086	0.38127174
Knee pain	1.098	0.4188066	1.072	0.25753329

a. Polygenic score for each chronic pain using PRSice-2 with sex and PC1-PC5 as covariates.

b. Polygenic score for each chronic pain using PRS-CS with sex and PC1-PC5 as covariates.

Supplementary Table S4. Assessments of associations between PGSs and SD, controlling psychiatric conditions

Controlled psychiatic conditions	MCP[a]		CWP[b]		CIDP[c]		AR[d]	
	OR[c]	p-value	OR[c]	p-value	OR[c]	p-value	OR[c]	p-value
SD | Substance use disorders	1.424	1.03E-04	1.323	2.35E-03	1.279	8.09E-03	1.191	5.21E-02
SD | Anxiety	1.280	1.05E-02	1.285	9.93E-03	1.171	9.40E-02	1.280	8.50E-03
SD | Bipolar disorder	1.379	1.42E-04	1.433	5.62E-05	1.193	3.92E-02	1.176	5.45E-02
SD | Major depressive disorder	1.471	1.28E-05	1.447	8.41E-05	1.181	6.62E-02	1.211	2.82E-02
SD | Opioid use disorders	1.350	6.63E-04	1.362	6.34E-04	1.277	6.29E-03	1.213	2.61E-02
SD | Sleep related disorders	1.351	2.24E-04	1.374	1.28E-04	1.181	4.14E-02	1.251	6.18E-03

a. Multisite chronic pain
b. Chronic widespread pain
c. Chronic inflammatory demyelinating
d. Monoarticular arthritis
