# Supplementary material for: Genetic risk of chronic pain conditions associated with risk of suicide death through an integrative analysis of EHR and genomics data
Source: Transl Psychiatry. 2026 Feb 16;16:117. doi: 10.1038/s41398-026-03861-6 (PMC12949045; doi:10.1038/s41398-026-03861-6)
Supplement: Supplementary file 3 — Supplementary Methods [file 41398_2026_3861_MOESM3_ESM.docx]

**Supplementary Methods**

**Specific types of pain**

Chronic inflammatory demyelinating polyneuropathy (CIDP) is an autoimmune peripheral neuropathic disorder associated with inflammation of nerve roots. CIDP impairs motor and sensory functions of upper and/or lower limbs, often symmetrically. The cardinal features include muscle weakness, atrophy, poor balance, paresthesia, and pain. The prevalence is estimated at about 5~9/100,000 people [1].

Monoarticular arthritis (AR) involves a single joint that is inflammatory and painful. It can have a range of pathophysiology including infection, trauma, rheumatoid arthritis, avascular necrosis, osteoarthritis, or gout, although up to 1/3 of patients do not present readily identifiable pathology [2].

Irritable bowel syndrome (IBS) is a functional gastrointestinal disorder, characterized by abdominal pain and discomfort and altered bowel habits. IBS is typically associated with visceral hypersensitivity and irregular gastrointestinal motility. It’s estimated to have about 6% prevalence rate in the US [3].

**Association of PGSs for control phenotypes with SD risk**

Statistical results derived from a GWAS study on hair color, which is likely not relevant to SD risk, were further examined as a negative control in our association findings. Given the public availability of GWAS results for four hair colors (e.g., light brown, dark brown, black, and red) [4], we estimated their PGSs and conducted analyses, employing methods consistent with our primary analysis, to assess their association with SD risk.

**References**

1. Dimachkie MM, Barohn RJ, Katz J. Multifocal motor neuropathy, multifocal acquired demyelinating sensory and motor neuropathy, and other chronic acquired demyelinating polyneuropathy variants. *Neurol Clin* 2013; **31**(2)**:** 533-555.

2. Keret S, Kaly L, Shouval A, Eshed I, Slobodin G. Approach to a patient with monoarticular disease. *Autoimmun Rev* 2021; **20**(7)**:** 102848.

3. Almario CV, Sharabi E, Chey WD, Lauzon M, Higgins CS, Spiegel BMR. Prevalence and Burden of Illness of Rome IV Irritable Bowel Syndrome in the United States: Results From a Nationwide Cross-Sectional Study. *Gastroenterology* 2023; **165**(6)**:** 1475-1487.

4. Jiang L, Zheng Z, Fang H, Yang J. A generalized linear mixed model association tool for biobank-scale data. *Nat Genet* 2021; **53**(11)**:** 1616-1621.
